# Mechanisms of cellular fitness and cell competition: Towards an integrated view

**DOI:** 10.1016/j.ceb.2025.102571

**Published:** 2025-08-09

**Authors:** Jules Lavalou, Karyna Kulakova, Yogaspoorthi J. Subramaniam, Eugenia Piddini

**Affiliations:** School of Cellular and Molecular Medicine, University of Bristol, Biomedical Sciences Building, University Walk, Bristol BS8 1TD, UK

## Abstract

Cell competition is a fundamental mechanism of tissue quality control that enables the selective elimination of less fit, mis-specified, diseased or aged cells. By shaping tissue composition, it plays a critical role in development, organismal health and a wide range of physiological and pathological contexts, including cancer. As its biological significance continues to grow, elucidating the molecular mechanisms underlying cell competition is essential for advancing our understanding of tissue biology, disease progression and future therapeutic strategies. In this review, we highlight recently identified, evolutionarily conserved pathways that govern cell competition through metabolites and systemic signals, proteostasis and mechanical exchange. By integrating findings across species and pathways, we reveal how these distinct mechanisms may intersect and coordinate to determine competitive outcomes, providing a conceptual framework to inform and guide future research.

## Introduction

**Figure 1:**
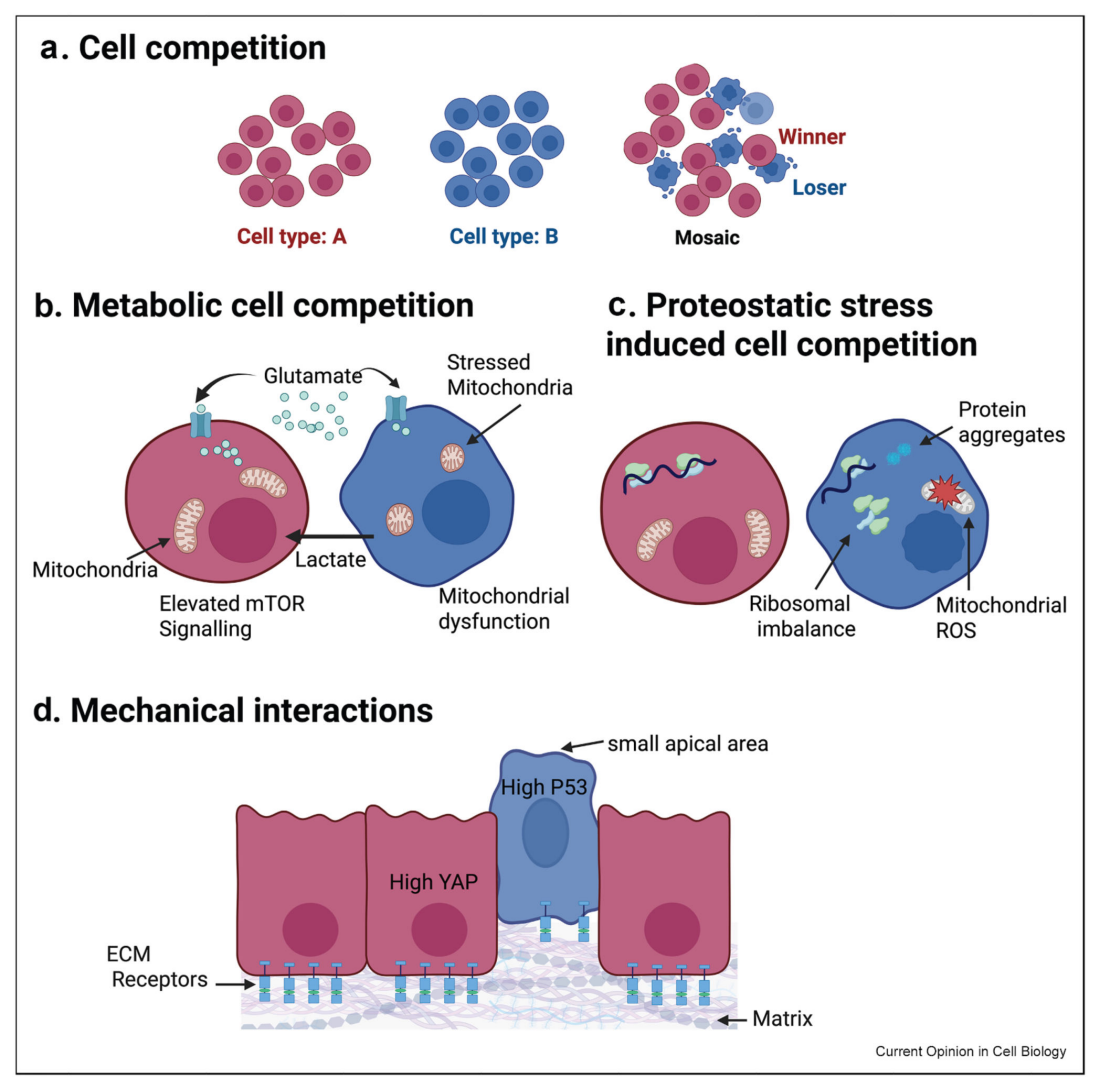
Mechanisms of cell competition. **(a)** Fit cells (in red) eliminate less fit neighbours (in blue) when in vicinity but otherwise remain viable on their own. **(b)** Metabolic cell competition arises when metabolites and metabolism differentially modify cell fitness between neighbours. **(c)** Cells with inherent loss of proteostasis become losers when challenged by cells with normal proteostasis. **(d)** Physical properties such as differences in sensitivity to crowding, in cell shape, in the ability to form cell–cell or cell–matrix connections, can influence the mechanical fitness of cells, leading to mechanical cell competition. Importantly, in each panel, we provide examples of modulators that have been identified in separate studies and contexts and we do not imply them to all work together. Created in BioRender.

Cell competition has emerged as a primary mechanism of tissue quality control. Exploited by cells to induce the elimination of neighbouring cells that are less fit, mis-specified, diseased [1] or aged [2], cell competition provides a mechanism for tissues to correct developmental errors [3] and delay the onset of ageing and disease [4,5]. Cell competition also plays a role in the dialogue between tumour and non-tumour cells, contributing to determine whether tumour masses expand or are, conversely, eliminated by normal neighbours [6], both in the initial phases of neoplastic transformation [7–10] and during metastatic relapse [11,12]. With the recent involvement of cell competition in myriad new processes, as diverse as injury repair [13,14], neural stem cell exhaustion [15] and species dominance in interspecies chimaera [16], the process of cell competition occupies centre stage to help us understand how cell interactions shape tissue biology and to offer strategies to modulate tissue fitness and composition in health and disease. To transition the field forward, from discovery research towards exciting therapy development, it is necessary to identify molecular handles that can be leveraged to control cell competition. Accordingly, here we review recently emerged mechanisms of cell competition, focusing primarily on pathways that could prove universal due to their conserved nature. We first introduce how metabolites and systemic signals, proteostasis and mechanical exchange individually modulate cell competition (Figure 1). Then we build on these concepts and integrate them together to highlight possible points of crosstalk between pathways. We discuss how these emerging connections suggest that seemingly distinct mechanisms of cell competition might in fact be interconnected at least in part.

## Metabolic and systemic regulation of cell competition

Cells synthesise or assimilate beneficial metabolites necessary for their survival. Conversely, they release/degrade metabolic products that would otherwise be toxic. One would therefore posit that cells with favourable ratio of beneficial/toxic metabolites should have a competitive edge in cell competition. Work from several groups is in broad agreement with this simple axiom, and this can be readily appreciated in early mouse embryos. Over one-third of mouse embryonic epiblast cells are naturally eliminated by apoptosis in early development and these cells display a loser signature, including low mTOR signalling, elevated P53 activity, low c-MYC and low expression of genes necessary for protein synthesis [17,18], suggesting that cell competition is a mechanism of cell quality control in early mouse embryos. Indeed, mouse embryonic stem (ES) cells with low c-MYC activity, with mutations in the BMP receptor (*Bmpr1a*^−*/*−^), with high P53 activity or with a tetraploid genome all behave as losers both *in vitro* and *in vivo*, in early mouse post-implantation embryos [17,19–21]. Intriguingly, loser cells with low mTOR, with *Bmpr1a*^−*/*−^ mutations or with tetraploid genomes, share a signature of impaired mitochondrial function, with reduced mitochondrial membrane potential, low oxidative phosphorylation (OXPHOS) and depleted intermediates of the tricarboxylic acid (TCA) cycle [18]. Importantly, rescuing their loser status rescues mitochondrial defects and conversely, introducing mitochondrial dysfunction can induce the loser status in epiblast cells. This suggests that differences in the ability to utilise metabolites for energy production can induce the loser status. Mitochondrial dysfunction has also been linked to the loser status induced by RasV12 expression in Madin—Darby canine kidney (MDCK) cells [22]. RasV12 leads to increased pyruvate dehydrogenase kinase 4 (PDK4), which depletes pyruvate dehydrogenase by phosphorylation, thereby diminishing mitochondrial membrane potential [22] and function. Importantly, PDK4 upregulation in RasV12 cells is necessary for their out-competition both in MDCK cells and in the mouse intestine [22]. While these studies indicate that reduced mitochondrial membrane potential can be a hallmark of losers, this is not always the case as earlier studies on *Drosophila* Myc^high^ cell, which are winners relative to wild-type cells, found that Myc^high^ cells display reduced mitochondrial activity and ATP production (low OXPHOS), and an increase in glucose uptake and lactate production, relative to wild-type cells [23]. Competition is also fuelled by a flow of lactate, which travels from losers to winners, sustaining their metabolic demand [24,25]. Glutamate signalling has also recently been identified as a key component of Myc^high^ cell competition; secreted by Myc^high^ cell, glutamate activates autocrine N-methyl-D-aspartate receptor (NMDAR, a glutamate receptor) signalling to support fitness. The study further shows that defective NMDAR signalling alone is sufficient to induce the competitive elimination of cells via autocrine tumour necrosis factor signalling [24], a major pathway involved in inflammation and cell death.

Given the multiple ways in which metabolism modulates cell competition, it is unsurprising that diet is emerging as a powerful modulator of cell competition. For example, in early models of neoplastic transformation, where Rasv12 cells are eliminated by normal neighbours through tumour-suppressive cell competition, obesity and high-fat diet act as tumour promoting, by tapering the elimination of RasV12 cells both *in vitro* and in mouse intestinal and pancreatic epithelia [26]. Another example is that of *scrib* clones, which are competitively eliminated by wild-type neighbours under normal conditions in *Drosophila* eye discs. However, diet-induced hyperinsulinemia, which strongly activates phosphoinositide 3-kinase (PI3K), allows *scrib* clones to overgrow by abrogating their loser status and cell competition, due to elevated activity of PI3K and its downstream effector mammalian target of rapamycin (mTOR) [27]. High dietary sugar and hyperinsulinemia can also boost the clonal dominance of Ras/Src-transformed cells in *Drosophila*. Whilst normal cells under hyperinsulinemia acquire insulin resistance, Ras/Src-transformed cells maintain insulin sensitivity, through upregulation of the insulin receptor, boosting their competitive growth [28].

Insulin availability shapes clonal dominance also in the oesophagus, where clones with high PI3K signalling display clonal advantage relative to normal cells [29]. There, supraphysiological insulin availability further increases their competitive fitness through PI3K signalling modulation.

Altogether, these studies show that MYC-, SCRIB-, RAS/SRC- and PI3K-mediated cell competitions are regulated by diet, hyperinsulinemia, metabolic availability and metabolic disorders, including obesity. This can have implications in modulating the outcome of cell competition, including during the expansion of precancerous lesions.

## Proteostasis loss and cell competition

In the recent years, regulation of proteostasis has emerged as a major factor impacting cell competition. The discovery came from investigations into the mechanisms of Minute cell competition, the first mode of cell competition ever discovered [30]. In Minute cell competition, *Drosophila* cells with single-copy mutations in ribosome genes behave as losers and are eliminated by wild-type cells. While it was long assumed that reduced translation, resulting from ribosome mutations, caused the loser status, this, however, is not the case, as rescuing protein translation in *Minute* cells does not rescue (rather worsens) Minute cell competition [31,32]. Instead, these studies identified a connection between proteotoxic stress and cell competition. *Minute* cells accumulate protein aggregates and activate numerous stress signalling pathways, among which many are modulators of proteostasis, such as proteotoxic stress, oxidative stress and the integrated stress response (ISR) [31–33], a major cellular stress response pathway that inhibits protein synthesis and induces stress response genes. This is evolutionarily conserved as defects in ribosome biogenesis induce protein aggregates and proteotoxic stress in several organisms, including in humans [32]. Importantly, treatments that ameliorate proteotoxic stress rescue cell competition. For example, mTOR inhibition, which promotes proteostasis by stimulating autophagy and diminishing protein translation, rescues proteotoxic stress, the ISR and the competitive elimination of *Minute* cells [31,32]. Notably, at least in *Drosophila*, proteotoxic stress marks cells as losers: inducing proteotoxic stress by overexpressing toxic protein aggregates [31], by increasing P-eIF2alpha levels [34–36] (a core component of the ISR, which at high levels can be aggregogenic [37]), by downregulating the E3 ubiquitin ligase *mahjong* [38] (VprBP in mammals) or by mutating *rer1* (a protein necessary for the assembly of multiprotein complexes in the endoplasmic reticulum) [39]; all make loser cells that are eliminated by wild-type cells through cell competition.

How does proteotoxic stress lead to the loser status? The simplest explanation would be that the response to proteotoxic stress earmarks cells as losers. However, this has proven difficult to demonstrate. For example, it has not been possible to establish if the ISR contributes to the loser status, as decreasing the ISR in *Minute* cells decreases their viability, indicating that this stress response is cytoprotective in *Minute* cells [31,32]. ISR is also observed in mouse embryonic stem cells with mitochondrial defects [18], which behave as competitive losers, and like in *Minute* cells, it has a cytoprotective role [40]. Activation of the oxidative stress response, which is often elicited in the presence of proteotoxic stress, has also been implicated in the loser status. Indeed, overexpression of Nrf2 (the transcription factor that orchestrates the oxidative stress response) can turn cells into losers [33] displaying a transcriptional signature similar to that of *Minute* and *mahjong* mutant cells [41]. However, Nrf2 may not account for the loser status of *Minute* cells as it is unclear if Nrf2 activity is in fact higher in *Minute* cells relative to normal cells [36]. In addition, Nrf2 inhibition increases lethality of *Minute* cells, suggesting that baseline Nrf2 activity in *Minute* cells plays a cytoprotective role [33]. Consistent with this, a recent study looking systematically at the role of Nrf2 target genes found that many contribute to the viability and/or competitive fitness of *Minute* cells [41]. The notion that oxidative stress modulates the loser status is supported by three more recent studies. Competing skin epidermal stem cells are eliminated more effectively via cell competition, if they have a depleted redox ratio [42]. Similarly, *p53* mutant clones in the oesophagus behave as winners if the tissue is exposed to low-dose ionising radiation, but the effect is abolished if mice are administered antioxidants [43]. Antioxidants also protect loser cell elimination in Zebrafish development by reactive oxygen species scavenging [44].

In *Drosophila* a key contributor to the loser status induced by proteotoxic stress is the transcription factor Xrp1. Xrp1 is upregulated in *Minute* and *mahjong* mutant cells [33,45] and in Nrf2 overexpressing cells [41] and is required for their competitive elimination [34–36,45,46]. Upregulated in response to proteotoxic stress, Xrp1 seems to contribute to the loser status by promoting proteotoxic stress, thus creating a feed forward loop and amplifying proteotoxic stress induced by *Minute* or *mahjong* mutations [34]. Indeed, in the absence of Xrp1, many of the stress pathways activated by ribosome gene loss are largely rescued [34–36]. Conversely, Xrp1 overexpression in wild-type cells is sufficient to inhibit protein translation and induce the ISR [34–36].

An important outstanding question is to what extent competition induced by proteostasis loss is conserved in mammalian cells. The role of Xrp1 orthologues in cell competition has not been tested in mammalian cells, although it has been proposed that in mammals Xrp1 functions may be mediated in part by p53 [47]. Relevant to this, downregulation of the mammalian orthologue of *mahjong*, VprBP/DCAF1, does induce cell competition *in vitro* [38]. As DCAF1 is required for ribosome biogenesis in mammalian cells [48], this could be evidence that proteotoxic stress leads to loser cell status in mammals. However, in *Dacf1*^*f/f*^ cells, nucleolar stress leads to p53 activation [48], which itself turns cells into losers as discussed in the next section. Thus, p53 elevation rather than, or in addition to, proteotoxic stress could earmark *Dacf1*^*f/f*^ cells as losers.

One limitation hindering progress in this field is the absence of suitable mammalian Minute models of cell competition. The mouse *Rpl24*^*Bst*^ mutation was thought to be homologous to *Drosophila Minute* mutations as *Rpl24*^*Bst*^ mice are viable but *Rpl24*^*Bst*^ cells are lost during embryonic development in embryos where wild-type cells are present [49]. However, this mutation leads to reduced protein synthesis but does not induce ribosome imbalance, contrary to *Drosophila Minute* mutations, and is therefore unlikely to induce proteotoxic stress [50]. Thus, underrepresentation of *Rpl24*^*Bst*^ cells may be due to reduced proliferation rather than active elimination. In sum, given that proteostasis-related signalling pathways are conserved in metazoans and are observed in some mammalian losers, it is tantalising to suggest their involvement in competition in mammals; however, this is yet to be directly demonstrated.

## Physical properties affecting mechanical cell competition

Mechanical interactions between cells can also lead to the selective elimination of a cell population, in a process known as mechanical cell competition [51,52]. The term mechanical cell competition was initially coined when it was found that cells with higher sensitivity to crowding or compaction are outcompeted by cells with lower sensitivity to mechanical inputs [51,52]. When sharing the same space, physical constraints allow winners to crowd losers into a level of compaction that is beyond their ‘comfort zone’, inducing apoptosis or cell extrusion [51]. However, since that initial discovery, a range of other external or cell-intrinsic variations―such as cell shape, tolerance to cell density, contractility or adhesion―have emerged as key properties in determining cellular fitness in conditions of mechanical stress. Thus, molecular changes in cells that impact one or more of these physical properties can turn cells into mechanical winners or losers. While mechanical cell competition, classically defined as crowding-induced elimination, was initially identified in kidney epithelial cells in culture [51] and in the *Drosophila* notum epithelium [52], competition driven by other mechanophysical cues has now been observed in several other contexts, including in human pluripotent stem cell cultures [53], in *Drosophila* spermatogonial stem cells [54] and in the mouse epidermis [2].

P53 activity is a key regulator of mechanical fitness. In MDCK cultures, cells with relatively high levels of p53 activity become hypersensitive to compaction induced by crowding, behaving as losers to cells with relatively lower p53 activity [51]. How p53 modulates a cells’ mechanical sensitivity is not yet understood and warrants further investigation. It is also unclear if all p53-mediated cell competition is induced mechanically. Indeed, p53 loss induces cell competition and/or clonal dominance in several different instances (skin, oesophagus, bone marrow and mouse embryonic stem cells to name a few [44,55–59]). However, whether these are mediated by mechanical interactions has not been determined, and in the case of mouse ES cells, it has been ruled out [60].

Ras^V12^-transformed MDCK cells are also eliminated via mechanical interactions. When co-cultured with wild-type neighbours, Ras^V12^ cells are subjected to mechanical compaction, leading to their extrusion [61]. In this case, compromised cellular adherens junctions cause HRas^V12^ to be compressed by surrounding normal cells [62]. Compressive forces are initiated by cell surface recognition involving innate immunity components. In both MDCK cells and in HaCat human keratinocyte-derived cells, leukocyte immunoglobulin-like receptor B3, expressed in wild-type cells is activated by interaction with MHC class I receptors expressed on HRas^V12^ cells, triggering contractile activation of the SHP2/ROCK axis and creating the extrusion force to purge Ras^V12^ cells [63]. The effects of Ras activation, however, are context-dependent as high-Ras activity makes cells winners rather than losers in the *Drosophila* notum. High-Ras mechanical winners outcompete wild-type cells through compaction-induced ERK downregulation in wild-type cells, which leads to activation of proapoptotic factors [64]. Mechanical cell competition has also been observed in human pluripotent stem cell cultures, where aneuploid variants emerge spontaneously and show clonal advantage, mediated by mechanical cell competition. Here, the key mediator of mechanical fitness is YAP activity. Through changes in actomyosin contractility, high YAP endows cells with heightened resistance to crowding, increasing mechanical fitness [53]. High YAP also confers winner status in mouse liver cancer [8] and in *Drosophila* epithelia [65]. A key outstanding question is whether in these contexts, increased competitive fitness is also mediated by mechanical means.

Cell shape and geometry have also recently been shown to feed into mechanical fitness. Cells with a small apical area relative to neighbours are prone to be eliminated from epithelial tissues in the *Drosophila* notum. This too converges onto YAP, via modulation of the upstream Hippo signalling, which is activated in cells that have a small apical area, leading to YAP inhibition and cell elimination [66].

The strength by which cells adhere to the extracellular matrix (ECM) can also result in cells mechanically outcompeting their neighbours, particularly in competition for access to the niche. For instance, *Drosophila* germline cell competition arises from differences in the ability of cells to modify and bind to the niche ECM. Germline stem cells (GSCs) mutant for the transcriptional regulator *chinmo* remodel the stem cell niche by secreting the ECM proteoglycan component Perlecan. At the same time, *chinmo* ^−*/*−^ GSCs upregulate Dystroglycan, which binds extracellular Perlecan, allowing strong retention in the niche. As a result, neighbouring wild-type cells with relatively lower Dystroglycan expression are outcompeted by losing access to the niche [54]. In the mouse epidermis, one of the few instances where YAP upregulation is causative of a loser rather than a winner status, this is also mediated by differential adhesion to the ECM. Skin grafts of YAP-activated cells fail to survive transplantation to wild-type mice, whereas successful engraftment and transformation occur when grafts are transplanted into genetically matched YAP-activated recipients. This is due to YAP-mediated type XVII collagen downregulation [67]. Indeed, differential expression of the COL17A1 gene in mouse basal keratinocytes *in vivo* or human keratinocyte cultures is sufficient to cause cell competition [2]. Hallmarks of cellular damage and ageing can also act as upstream modulators of this competition pathway as collagen XVII is degraded upon oxidative or genomic stress [2]. Importantly, in epidermal stem cells, genotoxic stress modulates cell competition through regulation of another cell adhesion component. The p53 activation downstream of genotoxic stress activates Notch signalling, which causes cells to be outcompeted through induction of differentiation and through downregulation of integrin beta 1, resulting in cell delamination [68]. As p53 has emerged as a regulator of competitive fitness in several other contexts (see discussion above and [17,19,43,51,55,57–60]), it will be important to determine if p53 activity modulates competitive fitness through regulation of ECM adhesion also in other contexts.

## Are there one or multiple mechanisms of cell competition?

Our understanding of mechanical and biochemical cell competition has clearly improved, highlighting new details and complexities. However, we are still far from addressing a key question: do cells use one or multiple mechanisms to compete? It seems that cells employ multiple fitness sensing mechanisms and multiple pathways to execute cell competition. What remains to be addressed is whether these converge onto common downstream effectors. Indeed, to a substantial extent, many of the pathways that we have discussed above for their role in cell competition are known to intersect and cross talk. For example, metabolism impacts on protein turnover and proteostasis [69]. Cell mechanics and metabolism reciprocally affect each other at multiple levels [70]. The consequences of these cross talks on cell competition have not been fully explored and constitute an interesting future research direction. It seems likely that some of these cross talks will offer opportunities for cell competition pathways to intersect and cross-modulate and for upstream modulators to control multiple mechanisms of cell competition at once.

**Figure 2:**
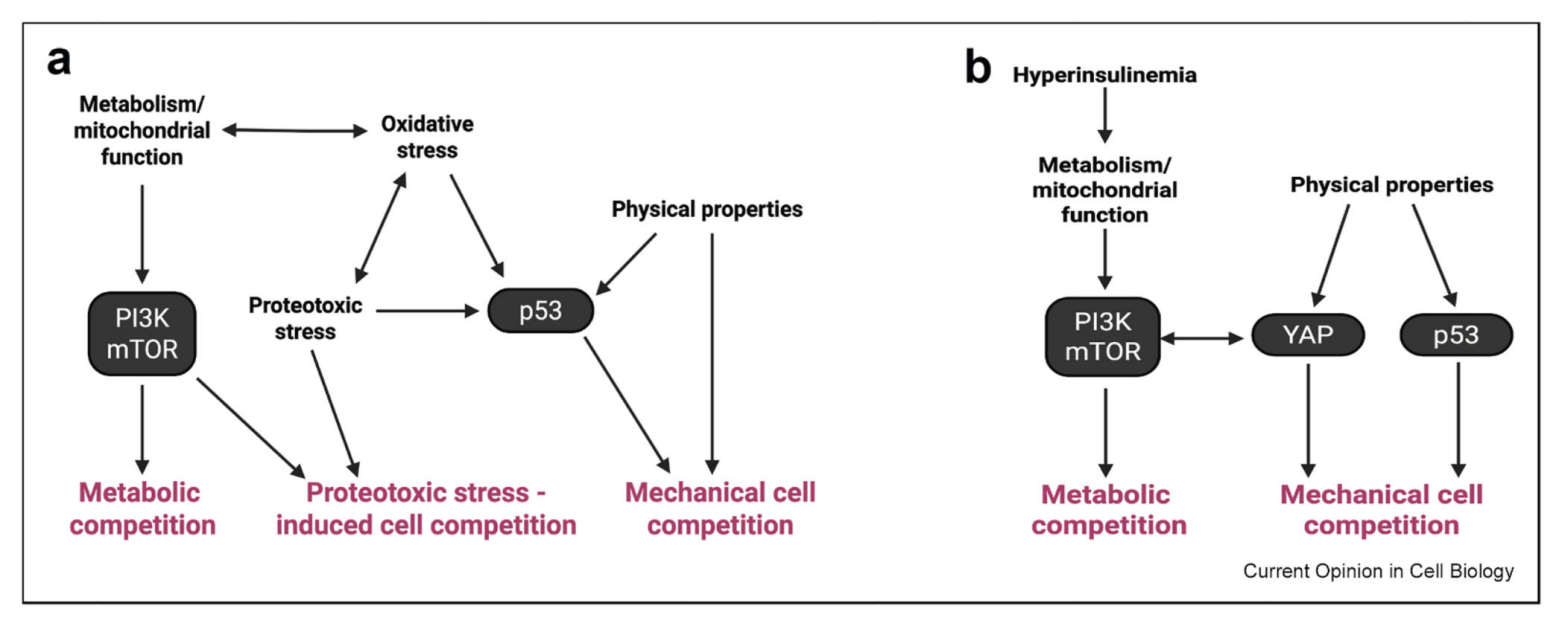
Interplay across different modes of cell competition. An integrated view of recently emerged modulators of cell competition highlights the high degree of interconnectedness across cell competition pathways. Bidirectional crosstalk between oxidative stress and proteotoxic stress and p53 likely connects multiple modes of cell competition downstream of a single input (2A). Bidirectional crosstalk between YAP and PI3K/mTOR is likely to result in connecting mechanical and metabolic competitions. Nodes of intersection (black ovals) across these pathways are emerging as central regulators with the potential to coordinate multiple fitness sensing mechanisms.

For example, p53 modulates mechanical fitness [51]; in turn, p53-mediated cell competition is modulated by oxidative stress [43], which itself is regulated by metabolic pathways [18] and by proteostasis [71], potentially linking these 3 types of competition (mechanical, metabolic and proteostasis-mediated) together (Figure 2a). Indeed, in mammalian cells, proteostasis-mediated competition is likely inextricably linked to mechanical cell competition as disruption of proteostasis e.g. via ribosome mutations or nucleolar stress, in addition to inducing the ISR, wills also lead to p53 activation [48]. Similarly, mechanical forces can activate p53 [51] and p53 is itself a regulator of metabolism [23], suggesting that these two pathways can crosstalk during cell competition.

As recently shown, cellular redox contributes to competitive fitness [42,43]. In turn, redox potential is heavily influenced by proteotoxic stress [72], mitochondrial function [73] and metabolites [74], potentially linking together oxidative status, proteostasis and metabolic cell competition (Figure 2a).

In the mouse liver, cancer cells induced by PI3K activation compete with peritumoural normal cells and this is modulated by YAP activity [8]. Given that, in other contexts, mechanical inputs modulate YAP signalling [70], it is likely that this competition is regulated by mechanical inputs and by pathways linked to mechanical cell competition. At the same time, as diet and hyperinsulinemia are known major regulators of PI3K activity [27,29], it is expected that these will also control competition here, providing yet another context where mechanical and metabolic competition could converge (Figure 2b).

Thus, when cells compete, multiple competition mechanisms are integrated together in determining competitive fitness and outcomes. This is also beautifully exemplified by the downregulation of collagen XVII in skin, which, as discussed, leads to keratinocyte loser phenotype. Collagen XVII indeed is downregulated by YAP activation [67] and by oxidative damage [2], thus receiving input from both mechanical and metabolic competitive signals.

In a reciprocal fashion, we are beginning to appreciate that external insults that acts as competition triggers can impinge on multiple competitive nodes: in the mammalian epidermis, genotoxic stress will activate both oxidative damage-induced cell competition (via collagen XVII downregulation) and DNA damage, which will lead to p53-induced cell competition [2,68], likely ensuring a robust response.

Evolutionarily, these emerging connections and intersections make a lot of sense as, ultimately, suboptimal cellular fitness needs to be integrated in a cell to provide robust and efficient cell elimination. Importantly, they also provide interesting opportunities for intervention as identifying key nodes where competitive fitness from multiple cell competition pathways is integrated can help understand how to leverage this process most powerfully for therapeutic interventions.

## Data Availability

No data was used for the research described in the article.
